# The genus *Macrobrachium* (Crustacea, Caridea, Palaemonidae) with the description of a new species from the Zaomu Mountain Forest Park, Guangdong Province, China

**DOI:** 10.3897/zookeys.866.32708

**Published:** 2019-07-24

**Authors:** Xiao-Zhuang Zheng, Wen-Jian Chen, Zhao-Liang Guo

**Affiliations:** 1 Department of Animal Science, School of Life Science and Engineering, Foshan University, Nanhai 528231, Guangdong Province, China Foshan University Nanhai China

**Keywords:** biodiversity, freshwater prawn, molecular phylogeny, morphology, oriental region, taxonomy

## Abstract

Evidence-based information is the foundation for addressing urgent global challenges in conservation and sustainable management of the freshwater biodiversity. The present study expands current knowledge of the genus *Macrobrachium* in Zaomu Mountain Forest Park, Guangdong Province based on the morphology, colouration, distribution, and molecular characteristics of *Macrobrachiummaculatum*, *M.inflatum*, *M.nipponense*, and an undescribed new species, *M.laevis*. *Macrobrachiumlaevis***sp. nov.** can be distinguished from its congeners by a combination of characters, which includes the shape of rostrum, smooth carapace, and male second pereiopod. *Macrobrachiumlaevis***sp. nov.** displays striking colour pattern, which could help to distinguish this species from other congeneric species in living specimen. Furthermore, the molecular characteristics of mitochondrial cytochrome c oxidase subunit I (COI) showed that this species has a sufficient interspecific divergence from its congeners.

## Introduction

The genus *Macrobrachium* Spence Bate, 1868 comprises 242 species and subspecies inhabiting fresh to brackish environments (De Grave and Fransen 2011). *Macrobrachium* species are native to all continents except for Europe (Holthuis and Ng 2010). Prawns of the genus *Macrobrachium* are widely distributed in China. They can be found in various water bodies, including lakes, reservoirs, rivers, ponds, streams, ditches, swamps, and subterranean waters.

Interest in *Macrobrachium* as a food has emerged throughout the world because of their delicious and unique flavor, and large size. *Macrobrachium* species have become an attractive food source, with good economic potential and high commercial interest in China. In addition, some colourful members of the genus *Macrobrachium* have attracted attention as ornamental pet prawn, and are traded in the ornamental fish market. Li et al. (2007) confirmed the existence of 33 species of the genus *Macrobrachium* in China. Recently, Guo and He (2008), He et al. (2009) and Chen et al. (2018) reported three new species from Guangdong Province, southern China. Furthermore, three new troglobitic species were reported in Guangxi Zhuang Autonomous Region, southwest China (Li and Luo 2001; Li et al. 2006; Pan et al. 2010; Lan et al. 2017; Cai and Ng 2018). A total of 39 species of the genus *Macrobrachium* have been recorded from China. The continued description of new species within *Macrobrachium* is a strong indication that there is still undiscovered species richness across the full taxonomic spectrum of *Macrobrachium* in China. The taxonomy of the genus *Macrobrachium* is mainly based on morphological characters, such as the relative length of the articles of the second pereiopods in fully developed males, rostrum shape and indentation, and colouration (Holthuis 1950; Chace and Bruce 1993). Some of these morphological characteristics have been proven highly variable within the species (e.g., rostrum shape and colouration). Furthermore, the second chelipeds in particular show a very high level of developmental and sexual variation, including allometric growth in males (Short 2004). This makes it a challenge to identify and distinguish different species, and almost impossible to identify juvenile, immature and adult female specimens. Thus, comprehensive molecular characterisations have become a crucial step towards resolving these longstanding taxonomic issues (Liu et al. 2007; Jose and Harikrishnan 2019).

The Zaomu Mountain Forest Park (22°43'–22°45'N, 112°45'–112°47'E) was rated as national 4A scenic area in 2012 (Zhang 2014). In recent years, tourism in the Zaomu Mountain Forest Park has been fast growing. The park has been reconstructed with newly climbing trestles, streams for drifting, and amusement facilities. However, the increasing exploitation of tourist resources has failed to recognise the conservation needs of different species that are found in this ecosystem. These may have negatively affected the species biodiversity of the prawn fauna in the scenic area. So far, the *Macrobrachium* fauna of the Zaomu Mountain Forest Park has not been accurately surveyed. To understand the diversity of the prawn fauna in the scenic area, intensive field surveys were carried out in the period from 2017 to 2018. The results of these field surveys have shown that there are four species of *Macrobrachium*; one of them is considered as a new species to science, *M.laevis* sp. nov.

## Materials and methods

### Study area

**Figure 1.**
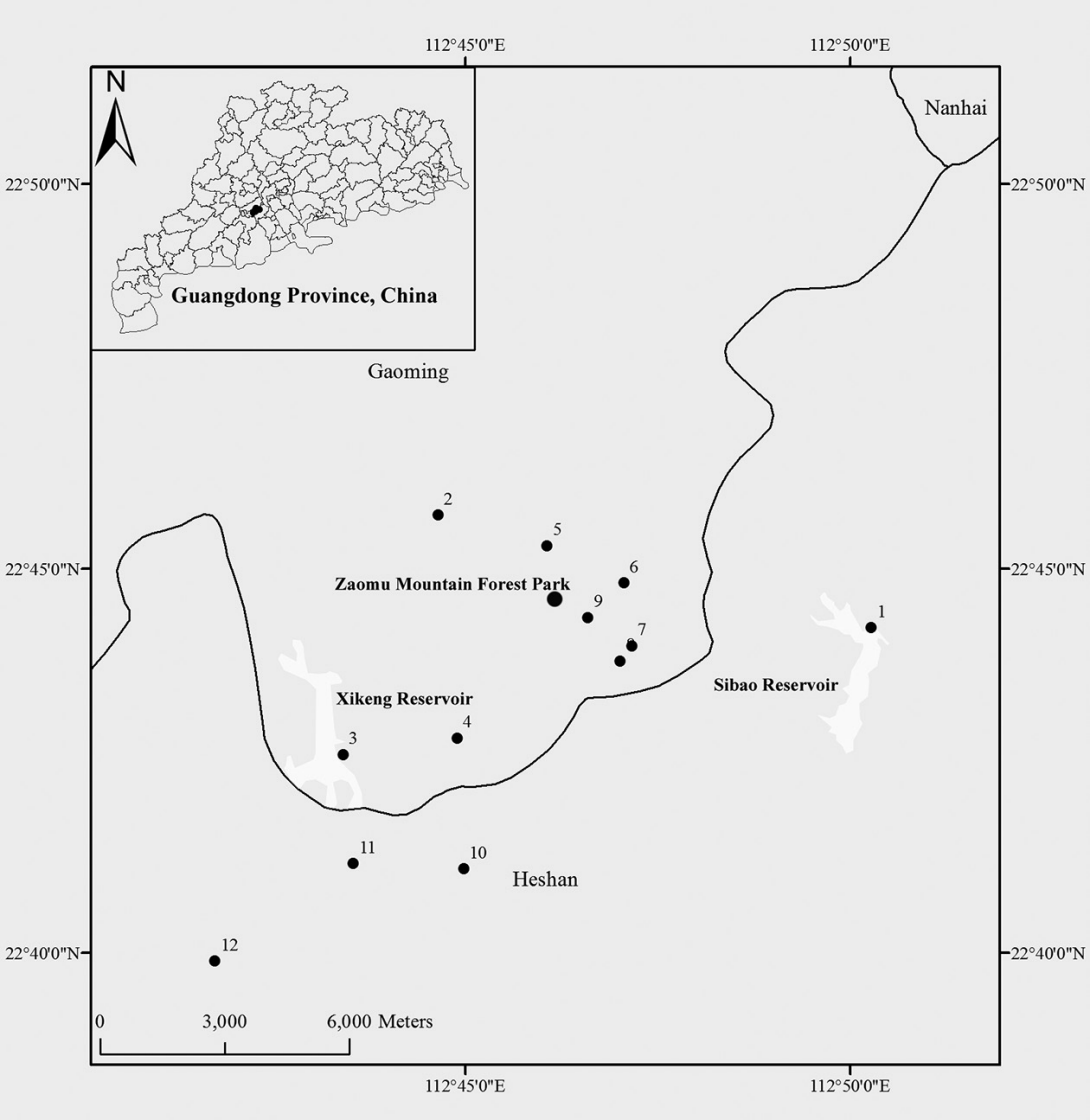
A schematic map of Guangdong Province, China. The expanded map shows locations of the Zaomu Mountain Forest Park and the 12 sampling sites.

The Zaomu Mountain Forest Park (112°45'–112°47'E, 22°43'–22°45'N) is located in the Youngmei Town, Gaoming District, 38 km West Foshan, Guangdong Province. The area stretches approximately 11 km from north to south and is 5 km wide. The total area is 55 square km. The main mountain peak is 804.5 meters above sea level, and is known as the highest peak of Foshan City (Zhang 2014). The Zaomu Mountain Forest Park has a subtropical maritime monsoon climate, which is warm and humid throughout the year. The mean annual temperature and precipitation are 22.5 °C and 1681.2 mm, respectively (Lu et al. 2003). There are many streams, ponds and reservoirs spread out the Zaomu Mountain Forest Park, and the Yangmei River runs through near the west. The locations of the sampling stations are shown in Figure 1.

### Collecting specimens

Samples were collected by a hand net with a mesh size of 0.8 mm. All specimens obtained were fixed in 95 % ethanol. Ethanol was replaced after 24 h with fresh 75 % ethanol. The drawings were made with the aid of a drawing tube mounted on an Olympus BX-41 compound microscope.

### Genetic analyses

Genomic DNA was isolated from the muscle tissue of the abdomen by using the Universal Genomic DNA Kit (Beijing, China). A fragment of the COI (619bp) gene was amplified with conventional polymerase chain reaction (PCR) using two primers LCO1490 (5’-GGTCAACAAATCATAAAGATATTGG-3’) and HCO2198 (5’-TAAACTTCAGGGTGACCAAAAAATCA-3’) (Folmer et al. 1994).

PCR cycling conditions consisted of a 3 min denaturation at 94 °C, followed by 35 cycles of denaturation at 94 °C for 30 s, annealing at 45–47 °C for 60 s, extension at 72 °C for 60 s, and a final extension at 72 °C for 5 min. PCR amplification sequences were obtained by sanger dideoxy sequencing (Applied Biosystems 3730), after verification with the complementary strand. The sequenced fragments were edited and aligned using Codon Code Aligner v. 8.0.2 (Codon Code Corporation, Dedham, MA, USA) and corrected by the naked eye. All sequences of this study have been deposited in GenBank Nucleotide Sequence Database (see Table 1 for accession numbers).

**Table 1.** List of locality, geographical coordinates, and GenBank accession numbers of eight palaemonid species used in the present study.

Species	Locality	Geographical coordinates	GenBank
			accession numbers
* M. dongaoensis *	Dong’ao Island, Zhuhai	22°01'39"N, 113°42'54"E	MK412789
* M. formosense *	Chancheng, Foshan	22°56'39"N, 112°53'41"E	MK412773
	Chancheng, Foshan	22°56'39"N, 112°53'41"E	MK412780
* M. inflatum *	Dongfang, Hainan	18°52'50"N, 108°59'29"E	MK412787
	Dongfang, Hainan	18°52'50"N, 108°59'29"E	MK412788
*M.laevis* sp. nov.	Gaoming, Foshan	22°43'60"N, 112°47'10"E	MK412774
	Gaoming, Foshan	22°43'60"N, 112°47'10"E	MK412775
	Heshan, Jiangmen	22°41'06"N, 112°44'59"E	MK412776
	Heshan, Jiangmen	22°41'06"N, 112°44'59"E	MK412777
	Gaoming, Foshan	22°43'60"N, 112°47'10"E	MK412781
	Gaoming, Foshan	22°43'60"N, 112°47'10"E	MK412782
* M. maculatum *	Gaoming, Foshan	22°45'18"N, 112°46'04"E	MK412770
	Gaoming, Foshan	22°45'18"N, 112°46'04"E	MK412771
	Gaoming, Foshan	22°45'18"N, 112°46'04"E	MK412785
	Gaoming, Foshan	22°45'18"N, 112°46'04"E	MK412786
* M. meridionalis *	Chancheng, Foshan	22°56'39"N, 112°53'41"E	MK412778
	Chancheng, Foshan	22°56'39"N, 112°53'41"E	MK412779
* M. nipponense *	Gaoming, Foshan	22°39'54"N, 112°41'45"E	MK412772
	Gaoming, Foshan	22°45'18"N, 112°46'04"E	MK412783
	Gaoming, Foshan	22°44'49"N, 112°47'04"E	MK412784
Outgroup			
* P. modestus *	Wulanhaote, Neimenggu	46°19'20"N, 121°54'45"E	MK412768
	Wulanhaote, Neimenggu	46°19'20"N, 121°54'45"E	MK412769

Six specimens of *Macrobrachiumlaevis* sp. nov. and 21 specimens of nine described species, namely *Macrobrachiummaculatum*, *M.formosense*, *M.meridionalis*, *M.nipponense*, *M.inflatum*, *M.dongaoensis*, *M.asperulum*, *M.fukienense*, and *Palaemonmodestus* were analysed in the prensent study. Sequences of *M.asperulum*and *M.fukienense* were obtained from GenBank for comparative and phylogenetic analyses. Two phylogenetic methods, maximum likelihood (ML) and neighbor-joining (NJ) were implemented. The best-fitting model for sequence evolution was determined by Modelgenerator (Goss et al. 2014) and selected by the Akaike Information Criterion (AIC). Pairwise genetic distances were calculated using the Kimura 2-parameter model with the pairwise deletion option in the MEGA 5 program. The phylogenetic tree was estimated using a NJ and ML method by MEGA 5 (Tamura et al. 2011), and the confidence level in the generated tree was obtained by using 1,000 bootstraps.

### Abbreviations

The following abbreviations are used throughout the text:

**alt** altitude,

**b** breadth,

**c** carpus,

**cl** carapace length, measured from the postorbital margin to the posterior margin of the carapace,

**f** finger,

**i** ischium,

**l** length,

**m** merus,

**p** palm,

**rl** rostral length, measured from the rostral tip to the postorbital margin,

**stn** sampling station,

**tl** total length, measured from the rostral tip to the posterior margin of the telson.

All measurements are in millimetres. Specimens were deposited in the Department of Animal Science, School of Life Science and Engineering, Foshan University (**FU**).

## Systematic accounts

### Palaemonidae Rafinesque, 1815

#### Genus *Macrobrachium* Spence Bate, 1868

##### Macrobrachium laevis sp. nov.

Taxon classificationAnimaliaDecapodaPalaemonidae

eec5975f-9a0f-509c-b008-b69931e16131

http://zoobank.org/E4A945BD-0988-40FB-B8E7-5199396E62D1

Figs 2 , 3

###### Material examined.

**Holotype**: Adult male (FU, 2018-01-15-01), tl 66.2 mm, cl 18.8 mm, rl 9.4 mm; a stream near the bamboo park, the Zaomu Mountain Forest Park, Guangdong Province China (22°43'60"N, 112°47'10"E, alt. 182 m, stn. 7), 15 January 2018. **Paratypes**: 7 males (FU, 2018-01-15-02) tl 45.0–61.1 mm, cl 11.8–16.4 mm, rl 7.2–9.3 mm. 14 females, 2 ovigerous females (FU, 2018-01-15-03), tl 39.8–61.5 mm, cl 9.9–17.3 mm, rl 6.5–9.3 mm, same data as for holotype. **Paratypes**: 2 males (FU, 2018-01-15-04), tl 32.1–48.8 mm, cl 8.0–14.2 mm, rl 5.0–8.0 mm. 1 female, tl 40.0 mm, cl 11.0 mm, rl 5.9 mm, a small stream near the Luohan hill, Heshan, Jiangmen City, Guangdong Province China (22°41'10"N, 112°43'33"E, alt. 140 m, stn.11), 12 May 2018. **Paratypes**: 8 males (FU, 2018-01-15-05), tl 43.3–51.2 mm, cl 11.8–14.4 mm, rl 8.5–10.1 mm. 14 female, 10 ovigerous females, tl 39.2–60.1mm, cl 10.1–16.5 mm, rl 6.5–9.6 mm, Longquan Gorge near Heshan, Jiangmen City, Guangdong Province China (22°41'6"N, 112°44'59"E, alt. 180 m, stn. 10), 12 May 2018.

###### Diagnosis.

Rostrum 0.51–0.71 of cl, tip slightly bent downwards, reaching to end of third segment of antennular peduncle. Rostral formula: 3-4+5-8/2-3 (usually 3), teeth equally spaced. Cephalothorax, abdomen, and second pereiopods smooth, without microspinules. Second pereiopods shorter than tl in both sexes; merus 1.0–1.2 times as long as the ischium; carpus 4.5–5.2 times as long as width, 1.2–1.4 times as long as merus and 0.8–1.0 times as long as palm. Palm not inflated, 4.8–5.3 times as long as wide. Movable finger 0.66–0.86 times as long as palm, without any gape when crossed. Fixed finger with one proximal tooth; moveable finger with two proximal teeth. All segments smooth, with only a small amount of spines along the lateral surfaces of the palm. Eggs large; size 1.1–1.4 × 1.5–1.8 mm diameter.

###### Description.

**Figure 2:**
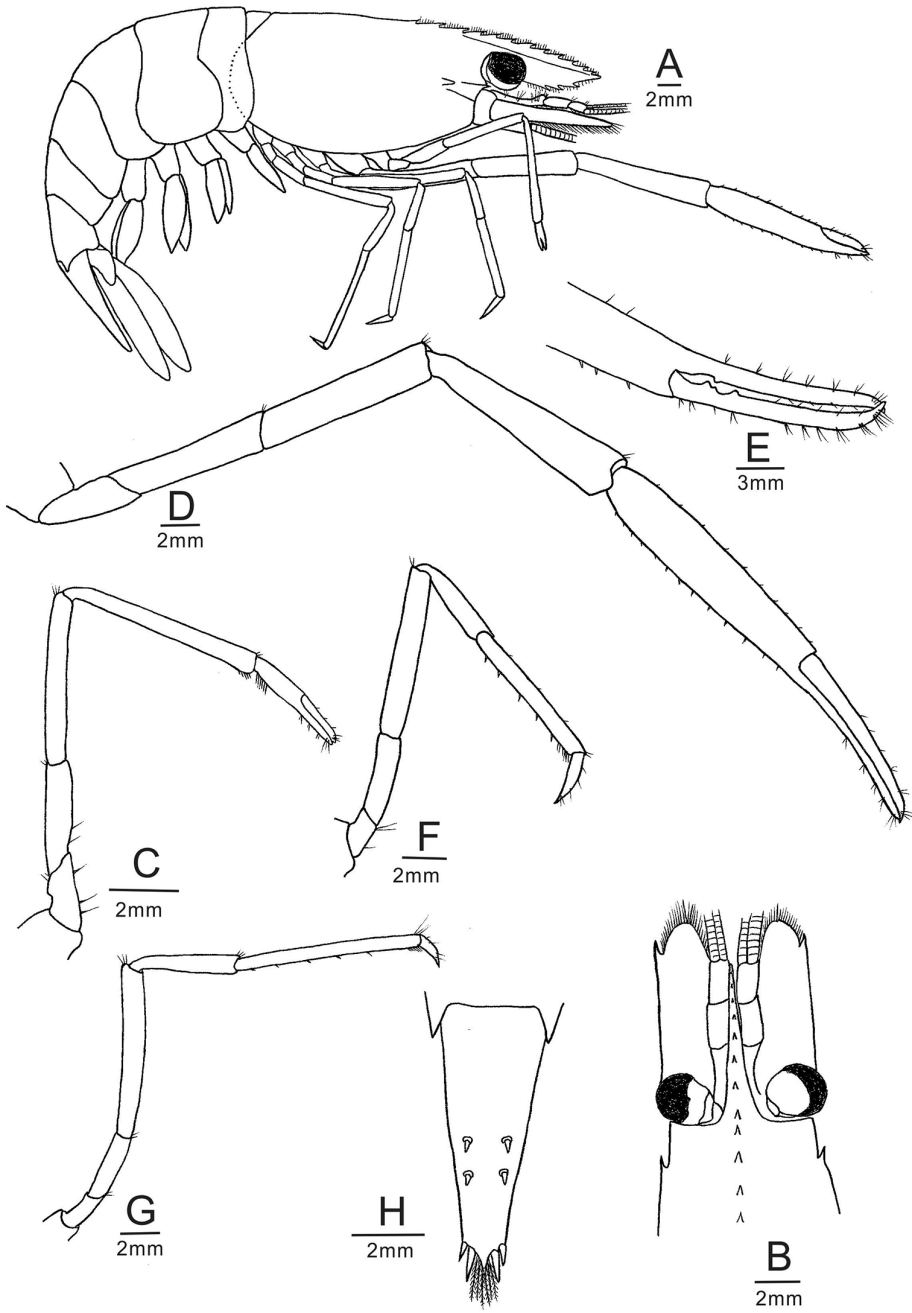
. *Macrobrachiumlaevis* sp. nov., holotype male (FU, 2018-01-15-01), cl 18.8 mm. **A** Entire animal, lateral view **B** cephalothorax and cephalic appendages, dorsal view **C** first pereiopod **D** second pereiopod **E** fingers of second pereiopod **F** third pereiopod **G** fifth pereiopod **H** telson.

Rostrum (Fig. 2A, B) rl 0.51–0.71 of cl, high, reaching downward to end of third segment of antennular peduncle. Dorsal margin with 8–12 teeth, three or four equally spaced teeth behind orbit; ventral margin with two or three teeth (usually three).

*Carapace* (Fig. 2A) smooth; antennal spine well developed, situated below lower orbital angle. Hepatic spine slightly larger than antennal spine, and slightly above level of antennal spine.

*Antennule* (Fig. 2A, B) bearing sharp stylocerite, reaching end of eye; anterior margin of basal segment distinctly convex, second segment 0.46 times as long as basal segment, 0.83 time as long as distal segment. All segments with submarginal plumose setae.

*Antenna* (Fig. 2A, B) bearing scaphocerite large, rectangular, 2.4–2.6 times as long as wide. Outer margin almost straight, ending with a strong spine, overreached by lamella.

*Mandibles, maxillulae, maxillae, first maxillipeds, second maxillipeds* and branchial formula typical for genus. *Third maxillipeds* with robust endopod and ischiomerus slightly bow-shaped, with rows of long simple setae on distal inner and outer margins. Carpus 0.70 times length of ischiomerus, with row of long simple setae on inner margin and sparse row of simple setae on outer margin; distal segment 0.83 times of penultimate segment, with long simple setae on inner margin. Exopod reaching distal end of ischiomerus, with plumose setae distally, basal with well-developed oval lateral plate; two arthrobranchs, one rudimentary, obscured by the larger one.

*First pereiopods* (Fig. 2C) slender, overreaching antennal scale by carpus; carpus 1.6–2.0 times as long as chela; fingers shorter than palm, 0.80–0.90 times as long as palm.

*Second pereiopods* (Fig. 2D, E) shorter than tl. Shape and segment ratios of left and right second pereiopods similar in both sexes, extending beyond the antennal scale by 1/2 of carpus; merus 1.0–1.2 times as long as ischium; carpus 4.5–5.2 times as long as wide, 1.2–1.4 times as long as merus, 0.80–1.0 times as long as palm; palm not inflated, 4.8–5.3 times as long as wide, movable finger 0.66–0.86 times as long as palm; fingers not gaping when crossed; fixed finger with one tooth at proximal, moveable finger with two proximal teeth; all segments smooth, only small amount of spines along lateral surfaces of palm.

*Third pereiopods* (Fig. 2F) extending to end of third segment of antennular peduncle by distal propodus; propodus 2.5–3.3 times as long as dactylus, with 5–7 spines on posterior margin; dactylus 5.5 times as long as wide, terminating in small claw.

*Fourth pereiopods* (Fig. 2A) extending to end of third antennular peduncle segment by distal propodus, somewhat similar to third pereiopods.

*Fifth pereiopods* (Fig. 2G) extending to end of third segment of antennular peduncle; propodus 3.4–6.5 times as long as dactylus, with 5–7 spines on posterior margin; dactylus 3.5 times as long as wide, terminating in small claw.

*First pleopods* of male with endopod of approximately half-length of exopod, slightly concave at inner margin, tip rounded, without appendix interna.

*Second pleopods* with well-developed appendix masculina, reaching middle of endopod, approximately twice as long as appendix interna with numerous stiff setae.

*Abdomen* (Fig. 2A) glabrous, smooth, pleura of first to third somites broadly rounded; pleura of fourth and fifth somites also rounded, but with almost rectangular posterolateral angle; sixth somite 1.2–1.4 times as long as fifth somite, 0.59–0.67 times as long as telson.

*Telson* (Fig. 2H) smooth, 0.46–0.61 times of cl, longer than sixth abdominal segment; dorsal surface furnished with two pairs of stout, movable, spine; posterior margin tapering regularly to a sharp point with two pairs of posterior a spine; numerous plumose setae present between inner pair of spine.

Uropodal diaeresis with a spine shorter than lateral angle.

Eggs large, size 1.1–1.4 × 1.5–1.8 mm.

###### Live colour patterns.

**Figure 3.**
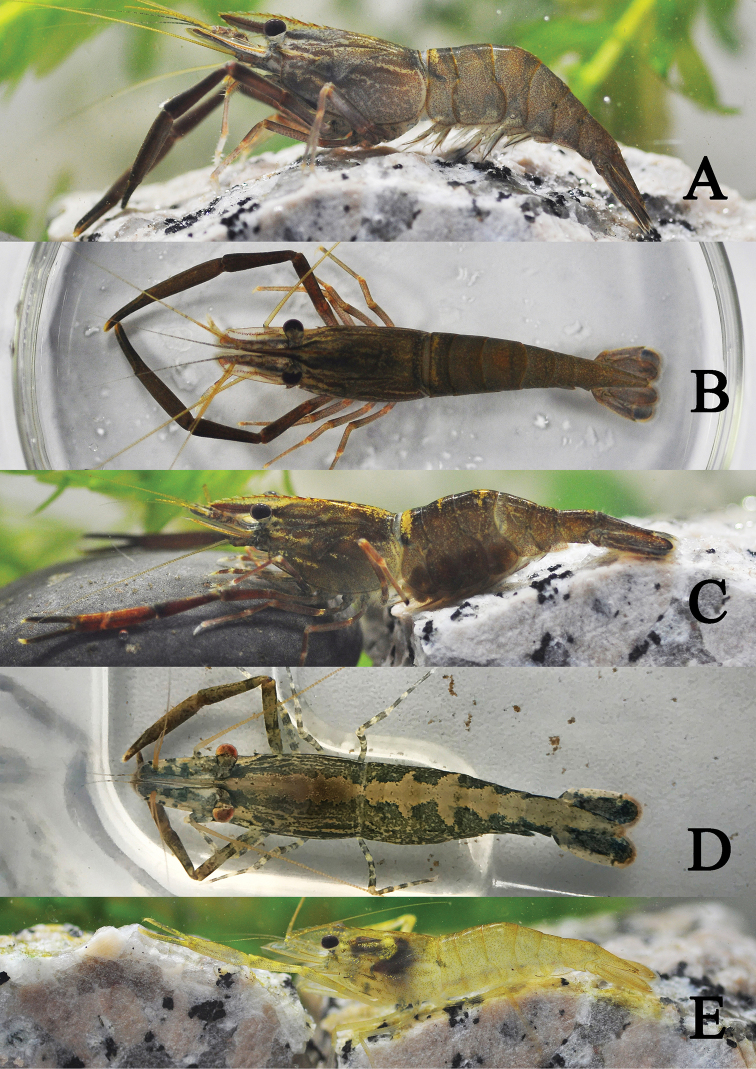
The colour of living *Macrobrachiumlaevis* sp. nov. **A** Lateral view of adult male **B** dorsal view of adult male **C** lateral view of ovigerous female **D** dorsal view of the fresh moulting female **E** lateral view of the immature male.

The juvenile was yellowish and semi-transparent (Fig. 3E); the adult male had a few indistinct longitudinal yellow stripes on the carapace, with one transverse yellow band on the first abdominal somite. All segments of the second pereiopods were golden (Fig. 3A, B). The ovigerous females had a pale yellow longitudinal stripe on the mid-dorsal surface from the rostrum to the tail, which extended to both sides of the abdominal somites. The palms of the third to fifth pereiopods had black and white rings (Fig. 3D). The eggs were brown (Fig. 3C).

###### Molecular phylogenetic results.

**Figure 4.**
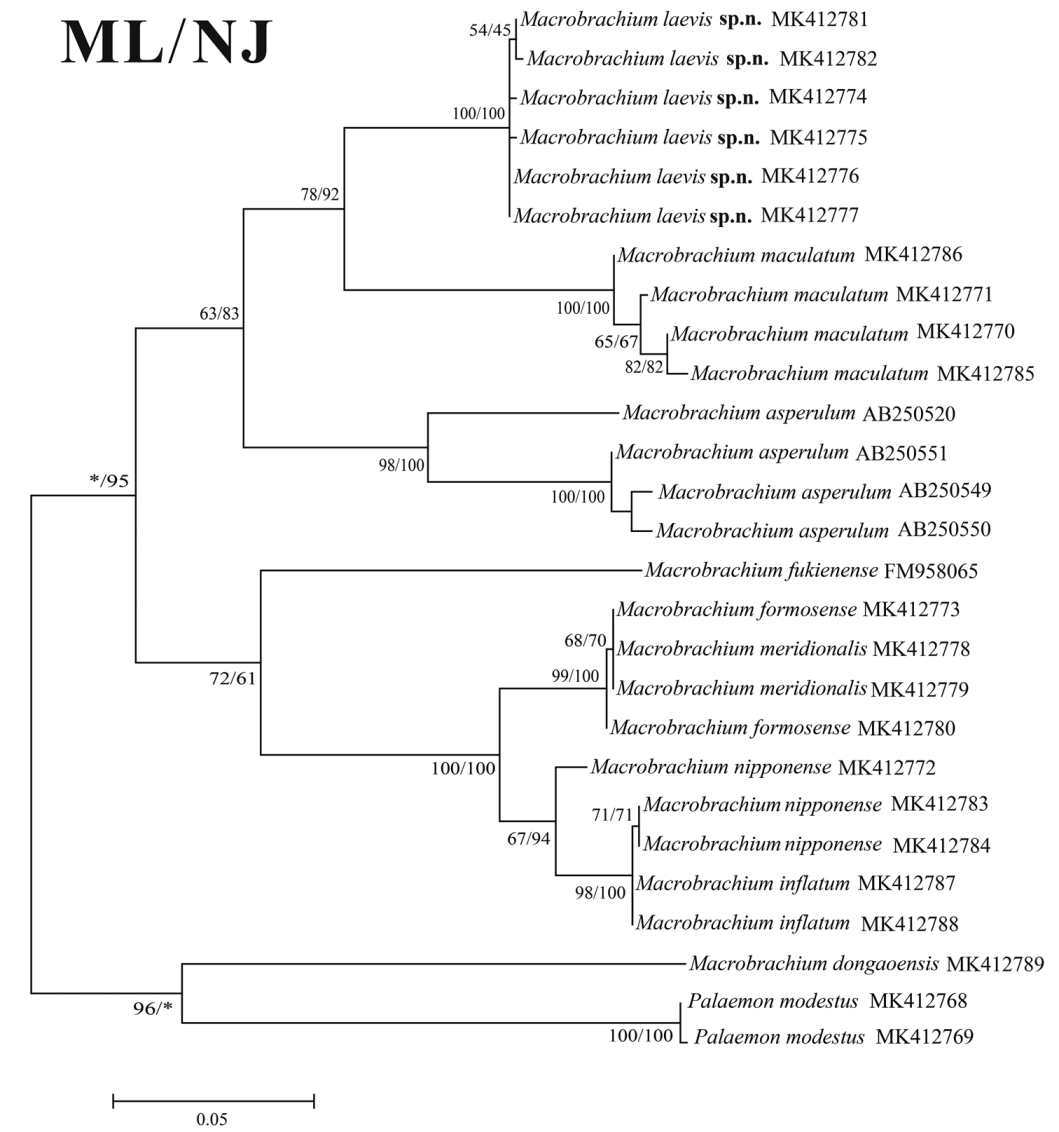
Phylogenetic relationships among *Macrobrachiumlaevis* sp. nov. and the other nine species, analysed by maximum likelihood (ML) and neighbour-joining (NJ) methods with *Palaemonmodestus* as the out-group taxa. Bootstrap values of ML (left) and NJ (right) are indicated above the branches of the clades.

Neighbour-joining (NJ) and maximum likelihood (ML) trees inferred from partial COI sequences (619 bp) from ten species of Palaemonidae, including the new species, are shown in Figure 4. *Macrobrachiumlaevis* sp. nov. is clustering with *M.maculatum* with high bootstrap support (96 % in ML tree and 96 % in NJ tree). Interspecific genetic divergence (K2P) among these ten species is summarised in Table 2. The pairwise distance was 0.15–0.62%. The new species was closest to *M.asperulum* (0.1475–0.1496) and *M.maculatum* (0.1095–0.1154), and the morphological characters supported this relationship. Moreover, the genetic divergence between *M.laevis* sp. nov., *M.inflatum*, *M.nipponense* and *M.dongaoensis* were 0.1558–0.1598, 0.1576–0.1617 and 0.2154–0.2218, respectively, supporting the morphological differentiation of the three species. Of the species analysed, *M.dongaoensis* was most genetically divergent from the new species (0.2154–0.2218).

**Table 2.** Pairwise genetic distance among nine *Marobrachium* prawn species based on the COI gene.

	**1**	**2**	**3**	**4**	**5**	**6**	**7**	**8**	**9**
(1) *M.maculatum*		0.138	0.169	0.166	0.167	0.167	0.192	0.171	0.103
(2) *M.asperulum*	0.138		0.162	0.160	0.171	0.171	0.203	0.151	0.135
(3) *M.inflatum*	0.169	0.162		0.014	0.053	0.053	0.197	0.146	0.148
(4) *M.nipponense*	0.166	0.160	0.014		0.050	0.050	0.193	0.144	0.145
(5) *M.formosense*	0.167	0.171	0.053	0.050		0.001	0.208	0.147	0.137
(6) *M.meridionalis*	0.167	0.171	0.053	0.050	0.001		0.208	0.146	0.138
(7) *M.dongaoensis*	0.192	0.203	0.197	0.193	0.208	0.208		0.207	0.182
(8) *M.fukienense*	0.171	0.151	0.146	0.144	0.147	0.146	0.207		0.155
(9) *M.laevis* sp. nov.	0.103	0.135	0.148	0.145	0.137	0.139	0.192	0.155	

###### Etymology.

Species name is derived from *laevis* (Latin) in reference to the smoothness of the segments of the second pereiopod, carapace, and abdomen.

###### Remarks.

*Macrobrachiumlaevis* sp. nov. shows close similarity with *M.maculatum*Liang and Yan 1980 regarding the ratios of various segments of the second pereiopods and in the rostral shape. *Macrobrachiumlaevis* sp. nov. can be distinguished from *M.maculatum* by the smooth second pereiopod whose margin of the palm has scattered microspinules (versus second pereiopod with microspinules on its whole surface); the second tooth of the movable finger placed on the proximal one-quarter (versus on the proximal one-fifth); the lack of papillae along the cutting edges (versus numerous papillae along the cutting edges); the finger slightly longer than the merus (versus the finger distinctly shorter than merus); the wider scaphocerite (2.4–2.6 times as long as wide) (versus 3.5 times); and ovigerous females carrying smaller eggs (1.1–1.4 × 1.5–1.8 mm) (versus larger egg sizes, 1.60–1.68 × 2.12–2.36 mm). *Macrobrachiumlaevis* sp. nov. is morphologically close to *M.asperulum*von Martens 1868 regarding the form of the rostrum and egg size. *Macrobrachiumlaevis* sp. nov. can be distinguished from *M.asperulum* by its smooth carapace and second chelipeds and lack of denticles on the cutting edges (versus with rough carapace and chelipeds, and the presence of approximately ten denticles on the cutting edges), and the second tooth of the movable finger at about proximal one-quarter (versus second tooth of the movable finger on the proximal two-fifths). *Macrobrachiumlaevis* sp. nov. superficially resembles *M.inflatum* Liang & Yan, 1985; however, *Macrobrachiumlaevis* sp. nov. can be distinguished from *M.inflatum* by its shorter rostrum with fewer dorsal teeth and reaching beyond the end of the third antennular peduncle segment, with 8–12 dorsal teeth (versus rostrum reaching beyond the scaphocerite, with 12–17 dorsal teeth); the palm of male second pereiopod being not inflated (versus inflated) and 4.8–5.3 times as long as wide (versus 3.5–3.6 times); the finger distinctly longer than merus (versus the finger as along as the merus); the ischium shorter than the merus (versus the ischium distinctly longer than the merus); and the ovigerous females bearing larger-sized eggs (1.1–1.4 × 1.5–1.8 mm) (versus 0.53–0.59 × 0.62–0.69 mm). *Macrobrachiumlaevis* sp. nov. is closely related to *M.fukienense* Liang & Yan, 1980. It is possible to distinguish *Macrobrachiumlaevis* sp. nov. from *M.fukienense* by the presence of more dorsal and postorbital teeth (8–12 dorsal and 3–4 postorbital teeth) (versus 7–8 dorsal and 1–2 postorbital teeth); the second tooth of the movable finger of the male second pereiopods on the proximal one-quarter (versus on the proximal half). *Macrobrachiumlaevis* sp. nov. is also closely related to *M.nipponense* De Haan, 1849. *Macrobrachiumlaevis* sp. nov. can be distinguished from *M.nipponense* by morphological characters of the male second pereiopods. The second pereiopods of *Macrobrachiumlaevis* sp. nov. are distinctly shorter than those of *M.nipponense*; the finger are distinctly longer than the merus (versus the finger shorter than the merus), and without setae on the cutting edge (versus the cutting edge with the long dense setae) (Fig. 3A, B versus Fig. 6A). It is possible to distinguish living *Macrobrachiumlaevis* sp. nov. from other congeners by its striking colour pattern (Fig. 3). Morphological differences between these congeneric species are presented in Table 3.

**Table 3.** Morphological characteristics of *Macrobrachiumlaevis* sp. nov. and the congeners.

	*M.laevis* sp. nov.	* M. maculatum *	* M. asperulum *	* M. fukienense *	* M. inflatum *	* M. nipponense *	* M. dongaoensis *
Rostrum							
Number of dorsal teeth	8–12	9–14	8–12	7–9	12–17	9–13	10–13
Number of postal orbit teeth	3–4	3–5	2–3	1–2	3–4	2–3	4–5
Number of ventral teeth	2–3	3–5	2–3	1–2	3–5	2–3	1–3
Ratio of RL/CL	0.5–0.7	0.6–0.7	0.6–0.7	0.6	1.0	0.6–0.8	0.5–0.7
First pereiopod Ratio of f/p	0.73–0.97	0.76–0.83	0.78	0.85	0.83–0.91	0.8	1.0
Second pereiopod							
Ratio of palm L/b	4.8–5.3	4.5–6.0	5.0–6.5	4.3	3.5–3.6	4.7–7.0	4.3–4.9
Ratio of f/p	0.66–0.86	0.62–0.78	0.5–0.6	0.4–0.5	0.82–1.0	0.6–0.7	0.69–0.78
Ratio of c/p	0.8–1.0	0.8–0.9	0.79–0.84	0.76–0.87	1.4	1.4	0.93–1.0
Ratio of c/m	1.2–1.4	1.1–1.3	1.3–1.4	1.1	1.4–1.5	1.6–1.7	1.1–1.4
Ratio of i/m	0.83–1.0	0.76–1.0	0.78	0.6–0.7	1.1	0.74–0.9	0.9
Ratio of f/m	≥1	<<1	<<1	<<1	=1	<1	>1
Microspinules on every segment	Smooth, except magrins of palm with scatted microspinules	All segments with	All segments with	All segments with	All segments without, except pout margin of palm with	All segments with	All segments with
Distribution of the second tooth of moveable finger	On the proximal 1/4	On the proximal 1/5	On the proximal 2/5	On the proximal 1/2	On the proximal 1/7	On the proximal 1/5	On the proximal 1/5
Eggs size (mm)	1.1–1.4 × 1.5–1.8	1.6–1.7 × 2.1–2.4	1.08–1.26 × 1.50–1.76	1.5–1.6 × 2.1–2.2	0.53–0.59 × 0.62–0.69	0.54–0.68 × 0.72–0.86	0.33–0.42 × 0.37–0.44
Scaphocerite l/b	2.4–2.6	3.5	2.8–3.2	2.5–2.8	3.4	2.7–3.1	3.4

###### Habitat.

**Figure 5.**
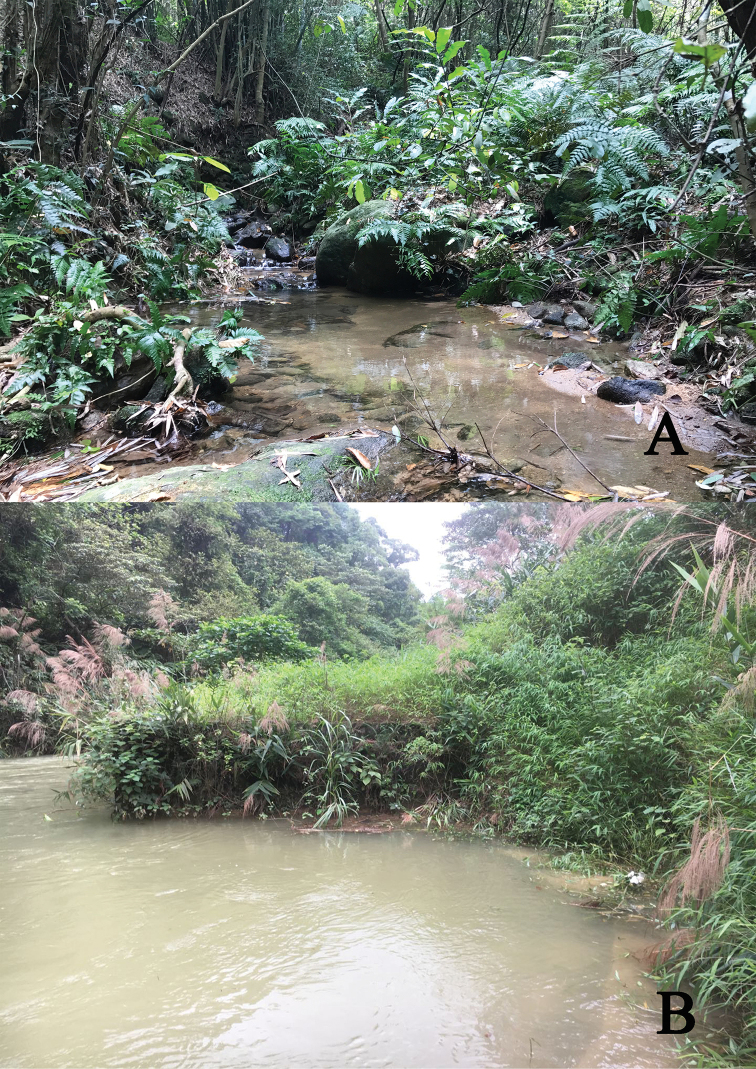
Habitats of *Macrobrachiumlaevis* sp. nov. **A** Stream near bamboo park, Zaomu Mountain Forest Park, Foshan City (type locality) **B** the Longquan Gorge, near Heshan, Jiangmen City. Both localities are situated in the Guangdong Province, southern China.

Specimens of *Macrobrachiumlaevis* sp. nov. were collected from two streams and a river. The stream was near bamboo park, the Zaomu Mountain Forest Park, Foshan City (22°43'60"N, 112°47'10"E, alt. 182 m, stn. 7) (Fig. 5A). This stream runs through land covered with a secondary forest, with beds of rock and patches of gravel. The stream width and depth were 2.0–3.5 m and 0.6–0.9 m, respectively, with fast flowing water. The water parameters of the stream at the time of collection (15 January 2018) were: temperature 13.8 °C, pH 7.0, dissolved ammonia nitrogen 0.2 mg/l, and dissolved oxygen 4.0 mg/l. The prawns were found at the bottom of the streams together with an atyid shrimp, *Caridinacantonensis*Yu 1938. The specimens were also collected from another small stream near the Luohan hill, Heshan, Jiangmen City, Guangdong Province (22°41'10"N, 112°43'33"E, atl. 140 m, stn. 11). The environmental conditions were very similar to the first stream. The water parameters of the stream at the time of collection (12 May 2018) were: temperature 25.6 °C, pH 6.5, dissolved ammonia nitrogen 0.2 mg/l, and dissolved oxygen 4.5 mg/l. Additional specimens were collected from the Longquan Gorge, near Heshan, Jiangmen City (22°41'6"N, 112°44'59"E, atl. 180 m, stn. 10) (Fig. 5B). It is a small river, with a total length of 6 km. The total drop of the river is 108 meters. The river resembles a jade belt and is deeply embedded at the bottom of the Zaomu Mountain. The river has flowing water, with rocks interspersed with sand patches at its bottom. The water parameters of the river at the time of collection (12 May 2018) were temperature of 26.1 °C, pH 7.0, dissolved ammonia nitrogen 0.2 mg/l, and dissolved oxygen 6.0 mg/l.

###### Distribution.

So far only known from the type locality and nearby localities in the Guangdong Province, southern China.

##### Macrobrachium nipponense

Taxon classificationAnimaliaDecapodaPalaemonidae

(De Haan, 1849)

5ff6f12a-6db7-538c-a95b-0db92c0ac455

Fig. 6A

###### Material examined.

Five females, tl 48.5–52.8 mm, cl 14.2–16.3 mm, 4 males, tl 51.3–65.9 mm, cl 18.0–25.6mm, Sibao Reservoir, Heshan, Jiangmen City (22°44'14"N, 112°50'17"E, alt. 84 m, stn. 1), 3 September 2017; 2 females, tl 48.5–50.4 mm, cl 15.2–16.4 mm, 1 male, tl 51.3 mm, cl 17.3mm, Lingshan Garden, Gaoming, Foshan City (22°45'42"N, 112°44'39"E, alt. 44.9 m, stn. 2), 17 May 2018; 3 females, tl 46.3–49.2 mm, cl 14.1–15.3 mm, 2 males, tl 51.3–61.4 mm, cl 18.2–23.4 mm, Xikong Reservoir, Gaoming, Foshan City (22°42'35"N, 112°43'25"E, alt. 22.4 m, stn. 3), 17 May 2018; 2 females, tl 42.5–44.1 mm, cl 13.2–14.6 mm, 4 males, tl 48.3–59.4 mm, cl 17.4–22.9 mm, Yangmei River, Gaoming, Foshan City (22°45'18"N, 112°46'04"E, alt. 49 m, stn. 5), 9 September 2017; 4 females, tl 41.4–50.3 mm, cl 12.3–16.3 mm, 3 males, tl 47.2–65.5 mm, cl 17.5–24.5 mm, stream near Hengkong Village, Gaoming, Foshan City (22°44'49"N, 112°47'04"E, alt. 72 m, stn. 6), 9 September 2017; 1 female, tl 42.5 mm, cl 13.2, 1 male, tl 48.3 mm, cl 16.7 mm, a stream near Datian Village, Gaoming, Foshan City (22°44'22"N, 112°46'36"E, alt. 56 m stn. 9), 17 May 2018.

###### Remarks.

*Macrobrachiumnipponense* were found in reservoirs, streams, rivers, and ponds of the Zaomu Mountain Forest Park. The species is native and broadly distributed throughout East Asia (i.e. China, Japan, Korea, Vietnam, and Myanmar), (Cai and Ng 2002; Li et al. 2007). *Macrobrachiumnipponense* was introduced into Singapore, Philippines, Uzbekistan, Iraq, Russia, Belarus, Moldova, and Iran (Chong et al. 1987; Alekhnovich and Kulesh 2001; Mirabdullaev and Niyazov 2005; Cai and Shokita 2006; De Grave and Ghane 2006; Salman et al. 2006). *Macrobrachiumnipponense* is commercially important in Guangdong Province where it is sold live in local fish markets, and is locally consumed at home and in restaurants as a special dish.

###### Colouration.

**Figure 6.**
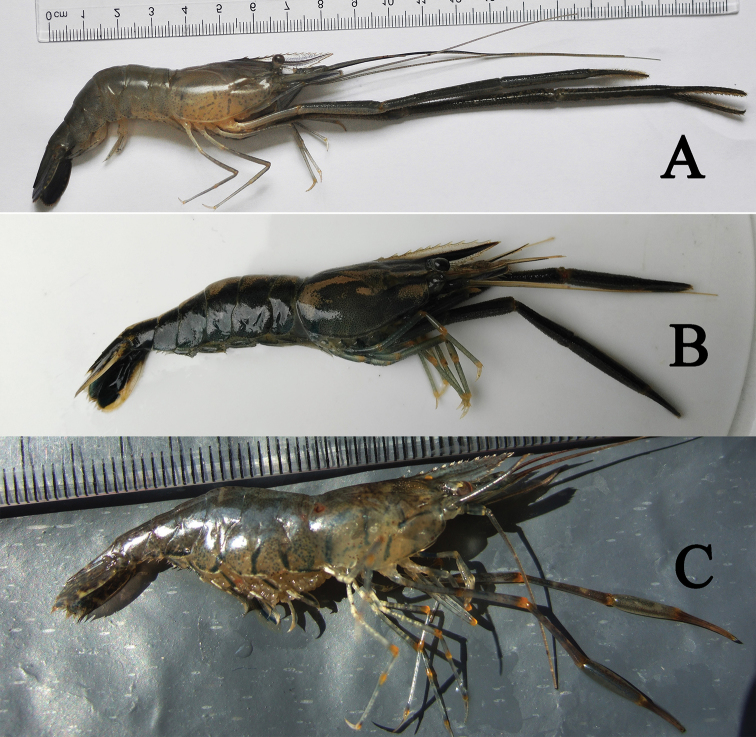
Photographs of *Macrobrachium* species. **A***M.nipponense*, living specimen, male **B***M.maculatum*, living specimen, male **C***M.inflatum*, living specimen, female.

The body has a lighter green and transparent colour, and the carapace has an M-shaped mark on the side (Fig. 6A).

###### Distribution.

China, Japan, Korea, Myanmar, and Vietnam.

##### Macrobrachium maculatum

Taxon classificationAnimaliaDecapodaPalaemonidae

Liang & Yan, 1980

22d3fc21-b39e-5041-b6f6-8a3fa198d5e1

Fig. 6B

###### Material examined.

Three females, tl 45.8–54.0 mm, cl 12.0–18.3 mm, 4 males, tl 35.6–75.8 mm, cl 9.6–19.8 mm, Yangmei River, Gaoming, Foshan City (22°45'18"N, 112°46'04"E, alt. 49 m, stn. 5), 9 September 2017.

###### Remarks.

The present specimens are consistent with the original description and illustration by Liang and Yan (1980) and Liu et al. (1990). This species is widely distributed in the southeastern China. *Macrobrachiummaculatum* has an economic importance and is usually found in the same habitat with *M.nipponense*. *Macrobrachiummaculatum* inhabits freshwater and has been found in rivers, reservoirs, and streams. This species seeks shelter among aquatic vegetation.

###### Colouration.

The body is very dark brown, the cephalothorax has diagonal yellow stripes, and the abdomen has large spots (Fig. 6B).

###### Distribution.

Southeastern China (Anhui, Hunan, Fujian, and Guangdong Provinces).

##### Macrobrachium inflatum

Taxon classificationAnimaliaDecapodaPalaemonidae

Liang & Yan, 1985

a695bca7-b228-5f57-9bd2-f2c933fbbfeb

Fig. 6C

###### Material examined.

Two females, tl 46.8–50.2 mm, cl 13.8–15.3 mm, 1 male, tl 52.1 mm, cl 14.2 mm, Qianlonggu, Gaoming, Foshan City (22°42'48"N, 112°44'54"E, alt. 124 m, stn. 4), 9 September 2017; 2 females, tl 45.5–51.0 mm, cl 13.4–16.1 mm, 3 males, tl 46.5–60.1 mm, cl 15.2–21.3 mm, Yangmei River, Gaoming, Foshan City (22°45'18"N, 112°46'04"E, alt. 49 m, stn. 5), 9 September 2017; 3 females, tl 40.5–54.3 mm, cl 12.5–20.6 mm, 2 males, tl 41.5–65.2 mm, cl 16.2–23.7 mm, Sibao Reservoir, Heshan, Jaingmen City (22°44'14"N, 112°50'17"E, alt. 84 m, stn.1), 17 August 2017.

###### Remarks.

Specimens were confidently assigned to the present species due to their inflated palm, the upturned rostrum and the rostral formula, as well as the ratio of the segments in the male second pereiopods. *Macrobrachiuminflatum* is usually found together with *M.nipponense*.

###### Colouration.

The body is translucent and light green. The rostrum is transparent to almost colourless. The cephalothorax has blue-black diagonal strips, and the abdomen shows blue-black transverse strips. The second pereiopods have transversal yellow bands on the merus and carpus. All joints of third to fifth pereiopods have transversal yellow bands. The eggs are yellow (Fig. 6C).

###### Distribution.

Southeastern China (Jiangsu, Anhui, Hunan, Guangdong, and Yunnan Provinces).

## Supplementary Material

XML Treatment for Macrobrachium laevis

XML Treatment for Macrobrachium nipponense

XML Treatment for Macrobrachium maculatum

XML Treatment for Macrobrachium inflatum
